# Animal Protection, Law Enforcement, and Occupational Health: Qualitative Action Research Highlights the Urgency of Relational Coordination in a Medico-Legal Borderland

**DOI:** 10.3390/ani12101282

**Published:** 2022-05-17

**Authors:** Dawn Rault, Cindy L. Adams, Jane Springett, Melanie J. Rock

**Affiliations:** 1 School of Criminology, Simon Fraser University, 8888 University Drive, Burnaby, BC V5A 1S6, Canada; dawn.rault@sfu.ca; 2 Department of Clinical and Diagnostic Sciences, Faculty of Veterinary Medicine, University of Calgary Clinical Skills Building, 11877 85 St NW, Calgary, AB T3R 1J3, Canada; cadams@ucalgary.ca; 3 Department of Community Health Sciences, Cumming School of Medicine, University of Calgary, Teaching, Research and Wellness Building, 3D10, 3280 Hospital Drive NW, Calgary, AB T2N 4Z6, Canada; 4 School of Public Health, University of Alberta, Edmonton Clinic Health Academy, ECHA 4-081, 11405 87 Ave NW, Edmonton, AB T6G 1C9, Canada; jane.springett@ualberta.ca

**Keywords:** animal welfare, communication barriers, criminology, ethical analysis, geography, law enforcement, One Health, occupational health, organizational case studies

## Abstract

**Simple Summary:**

In this article, we report on action research in the Canadian province of Alberta, based on forging alliances with officers who enforce federal, provincial, and municipal legislation involving animals. Some of these officers worked in rural areas, and others worked in urban areas. Some mainly enforced Alberta’s Animal Protection Act, while a few specialized in enforcing Canada’s Criminal Code. For the most part, however, participating officers had a mandate to enforce animal-related local bylaws. Such ordinances or rulebooks exist because Alberta’s Municipal Government Act allows local councils to enact legislation regarding domestic and wild animals, particularly as regards people’s health, property, safety, and welfare. Many professionals refer to policies such as these, which are deeply rooted in the western legal tradition, such as “animal control.” Our findings illuminate how animal-control policies and personnel can help to protect domesticated animals, not just people. Even so, our research highlights that animal-control policies as well as animal-protection policies routinely fail to protect officers who enforce legislation involving animals. Furthermore, whenever officers who enforce legislation involving animals work in unsafe conditions, this endangers human as well as non-human lives.

**Abstract:**

Across Canada and internationally, laws exist to protect animals and to stop them from becoming public nuisances and threats. The work of officers who enforce local bylaws protects both domestic animals and humans. Despite the importance of this work, research in this area is emergent, but growing. We conducted research with officers mandated to enforce legislation involving animals, with a focus on local bylaw enforcement in the province of Alberta, Canada, which includes the city of Calgary. Some experts regard Calgary as a “model city” for inter-agency collaboration. Based on partnerships with front-line officers, managers, and professional associations in a qualitative multiple-case study, this action-research project evolved towards advocacy for occupational health and safety. Participating officers spoke about the societal benefits of their work with pride, and they presented multiple examples to illustrate how local bylaw enforcement contributes to public safety and community wellbeing. Alarmingly, however, these officers consistently reported resource inadequacies, communication and information gaps, and a culture of normalized disrespect. These findings connect to the concept of “medico-legal borderlands,” which became central to this study. As this project unfolded, we seized upon opportunities to improve the officers’ working conditions, including the potential of relational coordination to promote the best practices.

## 1. Introduction

We prepared this article for a Special Issue devoted to the protection of animals, and our intention is to illuminate the contributions that can be made by officers who enforce “local laws” regarding animals. Local laws go by various names, including “bylaws” or “ordinances,” depending on the country and the languages spoken there [1]. Notwithstanding sociocultural and geographic differences, local laws regarding animals tend to have much in common [2]. First, local councillors and allied professionals should carefully word legislation pertaining to animals in relation to their jurisdictional mandates. Second, local councillors should ensure the adequate resourcing of programs, without which local laws regarding animals would remain moot. To name just a few examples, local authorities may choose—or not—to earmark resources for programs to inform citizens about local laws regarding animals, for personnel to investigate complaints, and for facilities where animals can stay and receive proper care following seizures [3,4].

Advocates for animals often hold diverging views on local laws regarding animals, and the scholarly literature reflects these differences. Perhaps the most contested element concerns the definition of animals as property in local laws, in keeping with western legal tradition [5]. Local laws such as these, and corresponding policies and programs, tend to be called “animal control” [6], often in contradistinction to “animal protection” [5,7]. By way of background, higher levels of government often allow local councils to adopt and enforce “local laws,” with a view to protecting people from harm and property from damage or outright loss. By implication, local councils may adopt and enforce legislation with clauses that protect people’s pets from harm by other people and their pets. In all such cases and clauses, however, the mandates of local councils to govern human and non-human lives pivots on defining domesticated animals in law as property [5]. Many activists and scholars have argued that any law that permits people to own animals cannot protect animals, in the end, because ownership is inherently harmful to non-human beings as well as to human beings. This tension has specifically been explored in the bioethical and animal labor literature [5,8]. Now, we respect the intention, but we are amongst those who cannot agree with this line of argument [4]. In what follows, we elaborate on our position through a project based on action research [9].

Before proceeding any further, however, we must draw attention to key insights from other scholars whose work helped us to develop this project. First, we subscribe to a broad understanding of legislation involving animals because we believe narrower definitions may, however inadvertently, undermine the interests of non-human beings. Following Huss (2009), we understand legislation involving animals to encompass all “legal issues that relate to or impact nonhuman animals” [10] (p. 1132). The scope of law enforcement under the umbrella of legislation involving animals is broad. Furthermore, under a predominantly complaints-based reporting system, both animal-protection and animal-control calls may be received and triaged by multiple different agencies [11]. As noted by Huss, this approach to the scope of legislation involving animals intersects with social movements concerned with animal rights and animal welfare, respectively. Huss acknowledges that her own scholarship has focused on laws that have impacted people with pets, whereas this project of ours concerns the impact of local bylaws on people and on the pets themselves [10]. The people in question include the legal owners of pets, as well as officers who have a mandate to enforce local bylaws, especially legislation adopted by local councils. Second, we take the view that outcomes for human and non-human beings may differ markedly, depending on how local laws become implemented, in practice. Put another way, the wording of local laws does matter, but so does implementation [12,13]. Third, we note that scholars have previously shown that local laws may, in practice, assist citizens and professionals in protecting animals in cases of hoarding [14], and a recent analysis focused on Canada’s legal framework [15]. This analysis became germane for our purposes, given that our project unfolded in the Canadian province of Alberta. Furthermore, sentinel cases in our project involved human behavior consistent with animal hoarding [16].

It was these sentinel cases involving animal hoarding that generated discussions regarding the work of officers in the medico-legal borderland. Broadly speaking, a medico-legal borderland is a place where the disciplines of medicine and the law meld to create new structures of knowledge and power [17]. Two decades have passed since Timmermans and Gabe published a seminal article that introduced the concept of medico-legal borderlands [17]. In coining this concept, they noted that the fields of criminology and medical sociology “…have developed largely independently of one another…” [17] (p. 501). This article reinvigorates discussions of medico-legal borderlands by exploring the work of local law enforcement at the intersection of animal welfare, community nuisances, and public health.

To begin to do this, we undertook an exploration of the experiences of officers who enforce legislation involving animals. We selected animal-related enforcement because there is a knowledge gap in our understanding of their unique role across the law enforcement continuum, from local laws to national statutes [18]. Officers who enforce local bylaws specifically experience multiple and often competing roles as agents of the law and public health professionals [15,19,20]. Taken in isolation, local laws and minor offences may not have significant impacts on individual or collective health, but their aggregate effects can have profoundly negative implications on the health of a community. Thus, the role of officers in mitigating such impacts merits our attention, and it is valuable to draw upon the seminal work of Timmermans and Gabe to explore this more fully [17].

Overall, we conclude that the work of officers in the medico-legal borderland warrants careful consideration. The need for coordination via the principles of relational coordination theory is highly relevant to the work of officers who enforce legislation involving animals. The lack of cooperation and coordination discussed in this paper highlights the importance of relational coordination to protect officers and animals [21,22,23]. The animals themselves may become much more vulnerable whenever advocates for animal protection and professionals in law enforcement and public health disregard officers who have a mandate to enforce legislation that defines non-human beings as property [5,15,24]. Disregard for such officers within health and legal systems puts their own health and safety at risk. To protect the animals, we must first protect the officers [24].

## 2. Materials and Methods

**Figure 1:**
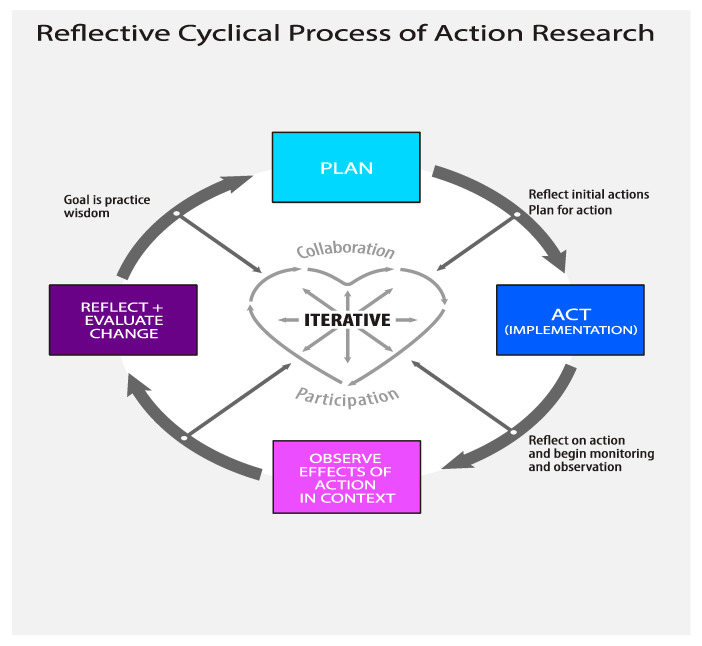
Reflective cyclical process of action research. Adapted with permission from [27], 2019, Rault.

The overall aim of action research is “practical wisdom” [25] (p. 17), which emerges through iterative processes of planning, acting, observing, and reflecting [25] (p. 17). This action research project involved a reflective cyclical process, utilizing a multiple-case-study design [26]. We illustrate our research process in Figure 1 [27].

Our project did not unfold as sequenced steps, but rather as a dynamic collaboration. Over a period of seven years, from 2012–2019, we worked with our research partners as a community of practice [28]. Near the beginning of the process, the first author established and strengthened connections with front-line officers and managers. These connections led to collaborations with two professional organizations that represent this workforce: The Alberta Municipal Enforcement Association (AMEA) and the Alberta Association of Community Peace Officers (AACPO). Through involvement with these organizations, our research has already resulted in relevant findings and informed policy discussions. As we learned about the challenges inherent to local law enforcement, the study evolved to focus on the officers’ own health and safety.

### 2.1. Learning from Officers and Managers

Action research is “…located in professional, cultural, and social contexts” [25] (p. 35). This study relied on immersive participant observation, including “ride-alongs”, which the first author documented in the fieldnotes and photos; in-depth interviews with officers and managers, which were ethnographic in style, meaning that the questions were tailored to the participants and built on preliminary analyses [29]; observations in court [30]; and legal and policy documents [26]. Of note, the first author has taught multiple post-secondary courses in justice studies, including field-based practicums involving local law enforcement. Hence, the first author’s familiarity with local law enforcement informed this project from start to finish.

Purposive sampling allowed us to consider the variations among officers working in different geographical and legal jurisdictions across the continuum of law enforcement. In total, we recruited twenty participants. Of these, ten served partly or entirely as managers, and nine officers had been enforcing legislation involving animals for a decade or more. Their respective mandates included the enforcement of legislation involving animals at federal, provincial, and local levels. In addition, some had professional experience with law enforcement at other levels than represented by their current appointments. The first author took care to recruit both male and female officers, as well as officers who worked in cities and in towns dotting rural areas. Additional officer demographics were not collected, but this matter warrants further discussion. In the larger centres, some of the participating officers specialized in enforcing legislation involving animals. For most of the participants, however, local bylaw enforcement varied shift-by-shift and seasonally. For example, the officers who enforced local laws tended to field more complaints about dogs being aggressive or roaming without adequate supervision in the spring, summer, and fall than in the winter months.

The participating officers were responsible for enforcing legislation involving animals in three cities and five towns. The smallest town had a population of 9386, while the largest city had 1,235,171 residents [31]. The first author personally conducted all the ride-alongs and the recorded interviews that form the backbone of this article. The first author also transcribed all the interview recordings, in a naturalistic style [32,33]. This study underwent a peer review in competitions for funding (grants #430-2016-00078, #130569, and #123380), and we received a research ethics certificate for this project (file #CHREB3-1101). As per that certificate, each participant signed a consent form after being briefed on the purpose of the study. Informed consent was obtained from all participants involved in the study. For the sake of confidentiality, we refer generically to “officers” in this article, or we refer to participants with pseudonyms in extended descriptions, or when gender identity seemed to shape what was said or conducted.

### 2.2. Understanding Working Conditions for Front-Line Officers

During the period of intensive fieldwork, descriptive fieldnotes were composed as an initial level of analysis. To complement the field notes, analytic memos were composed throughout as a second level of analysis. Analytic memos enabled the team to “reflect on and interpret what [we had] experienced and observed” in the field [30] (p. 100). Regular meetings of the research team informed our collective understanding of officers’ working conditions, including health and safety. We used QSR-NVivo 11.0 (QSR International, Burlington, MA, USA) to assist with organizing and interpreting the dataset [32].

Our approach to the thematic analysis was iterative and reflexive [33,34]. The process involved several overlapping phases as we sought to recognize, interrogate, and explore the officers’ working conditions, in general terms, and the enforcement of legislation involving animals, in particular. These analytic phases included: reviewing the dataset, with an emphasis on interview transcripts; generating initial descriptors as we began to recognize patterns; defining patterns and refining our descriptions of them; and producing summaries and elaborating on their implications for the participants and their counterparts. For us, thematic analysis seeks to represent patterns of acting, talking, and thinking that can meaningfully be expressed by a central organizing concept [35]. Additionally, in this study, the concept of medico-legal borderlands and relational coordination theory became central to our understanding of the work entailed by law enforcement.

### 2.3. Advocating for Officers in a Borderland Using Co-Created Knowledge

**Figure 2:**
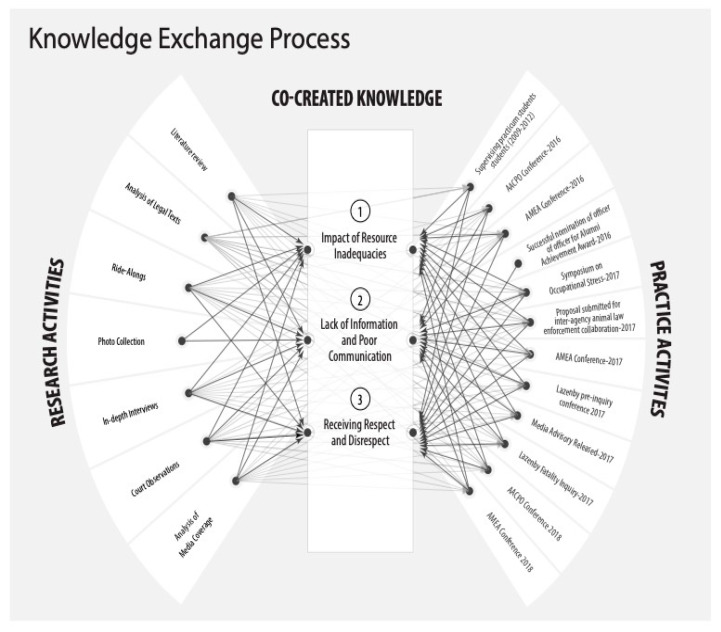
Knowledge generation and exchange. Adapted with permission from [27], 2019, Rault.

We aspired to a democratized process of research [9] (p. 257). Therefore, we sought to empower the participants and their colleagues throughout the research process, as illustrated in Figure 2. The legal status of animals creates tension that arises in practical terms and contributes to the precariousness of law enforcement. Our emphasis on empowerment reflects the marginalization of local bylaws and those on the front lines of enforcing these laws. Indeed, we came to regard the marginalization of local law enforcement as dangerous for all concerned, including officers, citizens, and the animals themselves. To date, we have made over twenty research presentations, including five invited presentations with policy makers in the audience. Whenever possible, to ensure recognition for their expertise, we shared the podium with officers. With each presentation, we directly challenged the status quo of unsafe working conditions for officers who enforce legislation involving animals at the federal, provincial, and local levels. Below, in presenting the findings from this study, we elaborate on how our research joined with advocacy efforts.

## 3. Results

During the ride-alongs and interviews, participating officers discussed the risks and rewards of law enforcement at length. Consistently, they said that law enforcement can contribute positively to community well-being and public safety, and they presented numerous examples drawn from their professional experience. This work included both officers who enforce local laws, and those whose mandate is exclusively to enforce animal-protection laws. The first author also witnessed the officers’ commitment to animals and people firsthand, during ride-alongs. This commitment positively impacts people, their pets, and more broadly, communities.

### 3.1. “I Guess You’re Not out Catching a Murderer”

Sadly, officers who investigate animal-related complaints often feel undervalued within the law enforcement hierarchy. One officer stated, “people want us doing this job, but they might not show the greatest respect for what ya do…I guess you’re not out catching a murderer…but you’re actually helping somebody.” As a counterbalance to feeling disrespected, officers felt valued when citizens provided positive feedback regarding the importance of their role in law enforcement. For example, Officer Laura worked hard to establish relationships in the community by using “positive tickets.” Positive tickets were coupons from local business for free goods, such as ice cream, chocolate, and coffee. They were handed out when community members were “caught in the act” by picking up their dogs’ waste, for example. Officer Laura explained that the use of positive tickets helped build a rapport with the community and enabled officers to interact with community members in a friendly way. Officer Laura remarked, “…you’re kind of like an ambassador for the town.” Officer Laura was one of seven participants who had previously worked as police officers. These officers brought a wealth of knowledge and experience to their work in enforcing provincial statutes and local laws, but they still experienced disrespect from the public and some law enforcement personnel. Feeling disrespected is not unique to law enforcement; tensions exist in interspecies and multispecies labour more generally [36,37,38,39].

Recognition from citizens and police officers for their skills in handling aggressive dogs also meant a great deal. In an incident involving an aggressive dog, Officer Dylan, who had two sons of his own, calmed the children while also collecting information from the parents. After the case was closed, Officer Dylan mentioned obtaining feedback from the family, saying, “…it’s nice to get feedback from the kids’ parents who email into your bosses to say how good of a job you’d done, thank you for all the help, and then I just think it’s part and parcel of the job, you’re there to do a job, that is my job to be a public servant.” Officer Dylan, who had previously worked as a police officer, told another story about an aggressive dog. That dog was loose in a community, terrorizing the residents. The dog’s behavior was so concerning that numerous police units were called to respond and the police helicopter was deployed to track the dog’s movements. With his expertise in dog handling, Officer Dylan humanely secured the dog. Until that point, the police officers thought they might have to shoot the dog. This incident demonstrates how the officer was deployed to protect the safety of the community, but ended up saving a dog that would have otherwise been destroyed.

### 3.2. “Like a Canary in a Coal Mine”

Officer Daniel’s employer was an animal welfare charity that typically receives over 1300 calls per year about animal abuse and neglect, and the handful of peace officers on staff diligently work with police services so that family violence involving animals can be prosecuted efficiently and effectively. One day, the first author was riding with Officer Daniel when he was tasked with picking up a deceased cat from a veterinary clinic. The cat had died as a victim of family violence. Given that domestic animals, such as cats, qualify as property under the law, Officer Daniel would subsequently hand over the cat’s body to the police of jurisdiction to serve as evidence in legal proceedings. Officer Daniel remarked, “Sometimes the animals are like a canary in a coal mine for domestic violence, or mental health issues, or other organized crime, that sort of thing, right?” By effectively collaborating with the police of jurisdiction, Officer Daniel recognized and responded to the human–animal violence link and had worked hard to foster a relationship of collaboration across multiple agencies [40]. This collaborative partnership has become the gold standard for animal cruelty departments nationally and internationally [24,40,41].

The animal welfare charity that employed Officer Daniel depends upon donations and grants to enforce Alberta’s Animal Protection Act. In principle, police officers can enforce this legislation, but in practice, peace officers employed by animal welfare charities have taken on most of the responsibility for enforcing the Animal Protection Act in Alberta [11]. All enforcement agencies operate with limited resources, yet the participants employed by animal welfare charities spoke of a need to be extremely prudent with spending. Whereas they could order a veterinary necropsy or request a tissue sample through a private laboratory to support an investigation into animal cruelty, such tests were only ordered when the officers and their managers felt confident of a conviction. Along these lines, the Edmonton Humane Society for the Prevention of Cruelty towards Animals backed away from law enforcement in 2019, requiring the City of Edmonton (population 972,000) Animal Control Peace Officers to quickly learn to respond to animals in distress or animals that have been abandoned [41,42].

The officers and managers who made this study possible spoke about their work in law enforcement with pride, yet they repeatedly expressed concerns about the risks entailed by this type of work to their own mental health and physical safety. Several units report to local councils, and some council members underestimate the risks associated with enforcing local bylaws. Similar problems exist amongst administrators. For example, a supervisor reportedly told a participating manager, “The day dogs carry guns, you’ll get body armor.” Another municipal manager, in describing his struggle to secure protective vests for front-line officers, told the first author, “…one of the first things that I did as a sergeant is I worked with my coordinator and put a lot of pressure on management to get us ballistic armor. Um, and body armor really was one of the first protective tools that we finally got authorized.” Discussions with officers and managers about occupational health and safety were dominated by references to the themes of resource inadequacies, information gaps, poor patterns of communication, and a culture of normalized disrespect for them in the law enforcement hierarchy (see Figure 2).

Below, to elaborate on these themes, we highlight two cases that received extensive media attention. Additionally, with reference to the ride-alongs and interviews conducted for this study, we show that these cases do not represent isolated incidents. All officers who enforce legislation involving animals, including local and animal-protection laws, face risks that are often not fully recognized.

### 3.3. Death in the Line of Duty

The death of an officer in August of 2012 profoundly impacted officers and managers involved in law enforcement, just as this study began. The officer’s name was Rodney Lazenby, and he died by strangulation while investigating a dog-barking noise complaint in rural Alberta, Canada.

Prior to taking up an appointment to investigate and enforce local laws in rural Alberta, Officer Lazenby had served thirty-five years with the Royal Canadian Mounted Police (RCMP). As a seasoned law enforcement professional, Officer Lazenby’s death in the line of duty could not be chalked up to inexperience. At the time, the accused and his over 30 dogs were living in a trailer parked inside a quonset. Eventually the accused was found not criminally responsible on account of a mental disorder [43].

In a recorded interview, one officer told the first author that Officer Lazenby’s death “…was a wake-up call for a lot of agencies, you know there’s so many peace officers working out there alone, working remotely, working without proper safety equipment.” Another officer’s employer had reportedly “…invested in some better communication equipment, so we now have the phones and the two-way radios, dispatch keeps a better eye on us at this point, we invested in some ballistic vests, so that we are personally more safe out in the field.” Officer Lazenby should have received mandatory officer training, access to two-way radio communication and intelligence sharing (including with dispatch), and worn standardized personal protective equipment. In addition, no officer should have attended the residence alone.

Officer Lazenby’s death was subject to a fatality inquiry, which occurred in June 2017. Behind the scenes, the first author helped to ensure that officers were represented at the fatality inquiry, could participate impactfully, and received recognition for their expertise in media coverage. Towards the end of the fatality inquiry, the president of one of the professional associations for officers chose to read a summary of the main themes from this study into the official record (see Figure 2). These themes captured the impact of resource inadequacies, lack of information and poor communication and the culture of normalized disrespect.

### 3.4. The Lazenby Fatality Inquiry and the Fraser Report

The fatality inquiry highlighted communication and information gaps between the local RCMP detachment and Officer Lazenby, even though Officer Lazenby had been a RCMP officer for much of his career. Officer Lazenby had investigated numerous complaints about the accused’s dogs and their barking, sometimes with RCMP officers in attendance [44] (p. 5). Meanwhile, the accused had been in contact with the RCMP detachment thirteen times. Unfortunately, the accused came to believe that Officer Lazenby was stealing his dogs. For instance, he called the RCMP detachment to complain after five of his dogs were reportedly released from their kennels [45]. The accused made numerous threatening calls to the local RCMP detachment, such as “I will be ready” and “you will pay.” As stated in the official report on the Lazenby fatality inquiry, “it is unknown if Officer Lazenby was aware of the threats” [44] (p. 5). All officers require reliable means of communication and information sharing with co-workers, their supervisor, and the police, including 911 dispatch. The local RCMP detachment have access to information about an accused person’s history, but peace officers attending a call know little about the occupants and whether extra safety precautions are required. Since communication between the two policing agencies was informal and information sharing with Officer Lazenby was limited, it is believed he was never fully apprised of the threats made against him [24].

Judge Fraser presided over the fatality inquiry, and his report contained recommendations regarding occupational health and safety for peace officers: (1) that no officer should attend a place where there is a known threat or interact with a person known to be mentally unstable without police back-up; (2) that all employers should know the whereabouts of their field officers at all times through central dispatch; and (3) that officers over the age of 40 years should be given additional time to complete the physical aptitude test [44]. Specific to the theme of information and communication, the judge wrote that “known risks should be distributed [to other agencies] and a risk assessment flagged not to attend alone” [44] (p. 10). Since the submission of this report, the Government of Alberta has acted on the recommendations for peace officers to receive backup support from police officers whenever a residence or a citizen presents known safety risks, and for a central dispatch service to monitor the locations of peace officers.

### 3.5. ”Flying Blind,” with “Spidey Senses” and “Funny Feelings”

Although Fraser’s recommendations are sound, in principle, several barriers exist to their implementation. First and foremost, officers who enforce legislation involving animals at provincial or local levels only cannot access the Police Reporting and Occurrence System (also known as PROS), so it is unlikely that they would be apprised of information pertaining to suspects’ mental health or any concerns about violence [24]. Some authorized officers, but by no means all officers, who enforce legislation involving animals have access to data in the Canadian Police Information Centre (also known as CPIC) [46,47].

Although some variation exists, for the most part, officers investigate and enforce legislation involving animals “blindly,” without any information about a residence or the persons who reside there. Officer Lily disclosed that she will often conduct an initial screen over the phone to gauge if there are any overt signs of aggression or mental illness:

“People who have been really abusive on the phone and threatening, or the complainant said, ‘Oh they’re drug dealers and the police are there all the time,’ that kind of thing. Cuz we’re not proxy to calls that the police have done, right? So we can go into a lot of places blind and not know what, if there’s any markers [concerns] on the address”.

Managers, meanwhile, reported that “flying blind” was a systemic problem. One manager said, “All I want to know [from police] is my officer going to be threatened by this person? Is this guy known to have weapons or be aggressive towards your [police] officers?” This manager worried that front-line officers would unknowingly interact with a person prone to violence or with a deep hatred for law enforcement of any kind. Another officer confirmed this concern, saying “…probably the biggest challenge that we run into…[is] access to information to keep our officers safe.” A colleague of that manager told a story about attending a property in response to a dog complaint, only to later learn from the police of jurisdiction that the property was under surveillance for drug and gang activity.

Inadequate information was especially problematic for officers who worked in smaller towns and rural areas. Officer Marianne, who must cover a large geographical area, often without radio or cell-phone coverage, told the first author that, in lieu of formal intelligence, she relies heavily upon her “spidey senses” to gauge the potential risk of a situation.

As another example, consider the working conditions of Officer Renee, who works for a privately-run enforcement agency that provides animal-control services to over 12 towns and communities. Officer Renee always worked alone, so she felt vulnerable, especially as a single parent of two small children.

During a ride-along, the first author accompanied Officer Renee on a drive out to a small town to deliver a ticket to a family residing in a mobile-home park. The complaint was that the family’s dogs were running at large and behaving aggressively towards neighbors. Officer Renee reported numerous interactions with this family, specifically for violations of the town’s animal-control legislation. In other words, this call was the latest of many complaints pertaining to the numerous dogs belonging to the family. Officer Renee described this family as “weird” and found communication to be a challenge. Members of the family had erected homemade no-trespassing signs explicitly warning people to stay off their property. The mobile-home itself was in disrepair and had materials stacked fully in the interior window wells, in a way reminiscent of hoarding as a mental disorder [48]. The family would not allow Officer Renee to enter the fenced area in front of their mobile home, which obliged her to use a doorbell located on the perimeter fence. When Officer Renee rang the doorbell, an adult son responded but refused to accept the violation ticket. Officer Renee then told the first author that she would request the back-up from nearby officers because she had a “funny feeling.” Even after Officer Lazenby was killed in similar circumstances, Officer Renee was still working alone without radio communication or any personal protective gear.

### 3.6. A “Near Miss”

Officer Lazenby’s investigation into dog-barking complaints occurred under the label of nuisance, a catch-all category in municipal legislation, which purports to protect the public interest [49]. Nonetheless, given that the “known facts leading to the death” included the observation that “the dogs were not being properly looked after” [44] (p. 4), the case that ended with Officer’s Lazenby’s death could have been investigated under the Animal Protection Act [50].

In addition, with more than 30 dogs in poor condition at the scene, animal hoarding seems likely to us in the Lazenby case. Hoarding disorder has recently gained recognition as a distinct disorder, and interventions into animal hoarding are amongst the most difficult and dangerous cases [48]. The not-criminally responsible verdict for Officer Lazenby’s killer, however, was rendered because he was actively psychotic at the time of the offence [24]. By strengthening communication between agencies, we can improve protections for officers, animals, and all the other individuals who may be involved in an investigation. We bring up these points about Officer Lazenby’s death as a backdrop for another high-profile case in law enforcement with a client who was mentally unstable.

Two years after the death of Officer Lazenby, in August 2014, three officers were threatened—verbally and physically—while investigating an animal hoarding case in Calgary, Alberta. At the time, the officers were inside the residence of Anthony and Christine Berry, a couple known to breed and show rabbits. The officers were acting on a tip from a concerned citizen, which had been lodged with the Calgary Humane Society, alleging animal abuse and neglect. One of the three officers was with the Calgary Police Service, while two were peace officers employed by the Calgary Humane Society. In addition, officers with the City of Calgary’s unit for animal services and bylaw enforcement were at the scene to assist with removing the animals inside the suspects’ home. Hence, animal-control officers could provide back-up support for the officers inside the home. This case provides an interesting exemplar for inter-agency coordination and collaboration.

Once the officers entered and began to uncover abject conditions of the rabbits and other animals in the home, Mr. Berry became agitated. He became verbally abusive with the officers, and then physically aggressive. The officers attempted to de-escalate the situation, but in vain. Rather, Mr. Berry produced an eight-inch rusted kitchen knife. Mr. Berry then stabbed the knife into the wall. Next, he grabbed an empty bottle, broke the bottle, and then stabbed himself in the neck. Meanwhile, Mr. Berry begged the police officer to shoot him [24].

Both Anthony and Christine Berry were later convicted under Alberta’s Animal Protection Act for causing their animals to suffer. In addition, Mr. Berry pleaded guilty to an assault charge under the Criminal Code. In conjunction with these proceedings, a forensic assessment of Mr. Berry’s mental state took place [24]. To the best of our knowledge, however, Mr. Berry did not receive a formal diagnosis of hoarding disorder.

Notwithstanding the abuse and distress entailed in the Berry case, the investigation qualifies as a “near miss,” especially in comparison with the Lazenby case and inquiry [51]. The “more hazardous a situation is, the more appropriate is its characterization as a near miss” [52] (p. 155). Without positive working relationships between police and peace officers, there could have been another fatality. In this case, a concerning phone call with Mr. Berry prior to attending the residence prompted a peace officer with the Calgary Humane Society to contact Calgary Police Service and inquire about backup. A police officer with expertise in legislation involving animals confirmed that the residence presented a safety concern for the peace officers. More specifically, the police officer had access to information systems showing that Mr. Berry had a history of violence and involvement with the criminal justice system, including charges of aggravated assault, confirming the presence of the human–animal violence link. Unfortunately, such positive working relationships and prioritization of legislation involving animals do not exist in all jurisdictions, but demonstrate the inherent benefits of inter-agency coordination and collaboration [53].

## 4. Discussion

The enforcement of federal, provincial, and municipal legislation involving animals takes place in a medico-legal borderland [17] for several reasons that our research has helped to illuminate. First, there exists ambiguity in jurisdiction amongst multiple levels of government, not least due to the ambiguous status of animals in law, as sentient beings, but also as privately-owned property [15]. This ambiguity can create tension across the law enforcement continuum. Second, legislation involving animals often apply to situations with both animal and human victims, such as child abuse and intimate partner violence [15,54], or that otherwise place both animals and people at risk, with dog-bite injuries and infections as common examples [6,55]. Despite this overlap, poor communication and coordination between agencies still exists. Third, animal abuse and neglect comprise cardinal symptoms for conduct and hoarding disorders, respectively [48,56]. Some jurisdictions are now collecting and sharing this data to better coordinate a response. Fourth, animal welfare charities participate in law enforcement by investigating allegations of cruelty towards animals, despite the challenges of being funded through charitable donations [11,41]. Fifth, unlike other medico-legal borderlands, veterinarians play important roles in law enforcement, for example, by substantiating allegations of animal abuse and neglect, or by assessing the level of risk or danger presented by a dog after a dog-bite incident. This duty to report animal abuse or neglect is now mandated by law.

Measures to safeguard officers’ health and safety are crucial to maximizing their potential in the medico-legal borderland of law enforcement. For officers, “the threat of physical harm is the most dramatic threat” that they face [57] (p. 32). Officer Lazenby tragically died as this study began, while the Berry investigation led to officer assault and self-harm by the main suspect. Along with physical danger and harm, emotional distress is perennial to law enforcement, and the effects can become debilitating [19,58]. Furthermore, the enforcement of federal, provincial, and municipal legislation involving animals can involve role strain, as officers must act as agents of the law and sometimes as health professionals [15,19,20,24].

Throughout this project, we explored ways in which Relational Coordination (RC) can help ameliorate some of the challenges experienced by officers who enforce legislation involving animals. RC plays a critical role in managing interdependence between people who perform tasks where high output, efficient collaboration predicated on respect, and safety are required [22]. In sum, RC is a “mutually reinforcing process of communicating and relating for the purpose of task integration” [22,23]. Plainly put, when people openly communicate and share goals, knowledge, and mutual respect, outcomes are beneficial for all stakeholders [22,23]. Although RC has traditionally been applied in the private industry, we are increasingly observing effective applications in youth services, educational settings, and long-term care, to name a few [22,23]. As a model for practice, RC can entail structural interventions that “support the changes with cross-cutting organizational structures” [22,23].

There are promising initiatives from around the world that conform to the propositions found in relational coordination theory. This paper explores two such programs to highlight the potential that exists in the field of law enforcement within the Canadian context. Specifically, Humane Canada have two initiatives that address one or more of the themes addressed in this paper relating to inadequate resources, intelligence, and a culture of normalized disrespect. First, the National Centre for the Prosecution of Animal Cruelty (NCPAC) “...is a community of Crown Prosecutors and allied professionals from across Canada who are working together in the service of the public interest to ensure that crimes against animals are prosecuted effectively and efficiently” [59]. The NCPAC actively engages allied professions, including veterinary and law enforcement professions, recognizing that prosecution is most effective when professionals across the spectrum work together [59]. This initiative fosters mutual recognition and respect across the law enforcement continuum and collaboration among allied animal professionals, consistent with the RC model. This program helps to create the structures that foster shared information systems and, in turn, benefit from shared accountability and rewards.

Related to this initiative, and also facilitated by Humane Canada, is the Canadian Violence Link Coalition [59]. The Coalition “…brings together professionals working to prevent and address violence against people and animals in more than ten Canadian sectors and is committed to advancing awareness, education, and training about the link between violence against humans and violence against animals. Its goal is to introduce violence prevention and intervention strategies across the country and to establish policies and practices that make our communities safer” [59]. The coalition acknowledges that violence against people and animals often co-occurs, and that officers are poised to introduce violence prevention and intervention strategies that cross species boundaries. This initiative is applicable to both the Lazenby and Berry case, and the broader discussion regarding the work of officers in the medico-legal borderland in that officers’ work transcends the boundaries of public health and criminal justice. These two initiatives demonstrate the potential that exists when we share goals and knowledge.

In order for collaborative practices to become normative, we have articulated the importance of recognizing and respecting the work of officers both in public health and across the law enforcement continuum. We believe positive and encouraging changes are happening that will improve the working conditions and recognition of officers across the spectrum of law enforcement.

Throughout this project, we sought to close the “knowledge to action gaps” that often persist, despite the best efforts of academics and practitioners [60] (p. 97). More specifically, we aimed to “…achieve changes to practices and to the contexts where these practices are located…” [25] (p. 39). In collaboration with the officers and their professional associations in our setting, we articulated recommendations to improve on working conditions, and, thereby, to increase public safety (see Figure 2). Specifically rooted in relational coordination theory, we recommended improvements in resource allocation, access to information, and communication amongst professionals [22,23]. It is notable that these recommendations directly influenced the Lazenby fatality inquiry. Following on from the Berry case, we assisted officers to create a business plan in support of a dedicated multi-agency unit to investigate allegations of animal abuse, keeping in mind that such cases may also involve child abuse, intimate partner violence, and mental illness [15,54,56,61,62,63,64]. This dedicated multi-agency unit is currently being run as a pilot program.

Overall, we sought to promote respect for the work entailed by local law enforcement. Yet, despite the legal requirements to protect workers, numerous agencies have yet to assess workplace hazards or to adopt appropriate procedures for local bylaw enforcement. Similar problems have been documented for officers employed by animal welfare organizations in Nova Scotia [65] and Ontario [58]. We hasten to add that the concerns raised about animal welfare charities as employers have systemic origins. Indeed, public funding for animal protection remains rare in Canada [66]. Compared with animal-protection work, even less attention has been paid to health and safety amongst animal-control officers. Yet, this work can be dangerous in the extreme, as underscored by Officer Lazenby’s death, as well as by the findings from fieldwork in the present study.

## 5. Conclusions

In this study, we explored the many risks and rewards involved in the enforcement of legislation involving animals, including both animal-control and animal-protection work. Whenever animal law enforcement is marginalized and poorly resourced and coordinated, vulnerability heightens for all concerned, including the officers and the animals themselves. These impacts ripple further into communities and neighborhoods.

The death of Officer Lazenby in the line of duty tragically taught us that when the working conditions for officers are unsafe, it endangers human and non-human lives. Although the Lazenby investigation originated as a barking complaint, it could have been any number of agencies that attended that residence. Because of the complex nature of officers’ work across the medico-legal borderland, increased communication and coordination within and between agencies is recommended to improve the desired outcomes for each organization.

The “near miss” story involving the Berrys examined the potential and power of inter-agency collaboration and cross-sectoral cooperation. The agencies involved in this case shared goals, knowledge, and, importantly, mutual respect. This reciprocity helped to facilitate timely communication, which likely prevented a more serious violent encounter. Since the Berry case, the agencies involved have formalized their partnership, serving as an exemplar for other jurisdictions looking to effectively coordinate.

Measures to safeguard officers’ health and safety are crucial to maximizing their potential in the medico-legal borderland of law enforcement and animal protection. We hope that this article will help to spur action on occupational health and safety in law enforcement, at all levels of government as well as in the charitable sector, throughout Canada and in other countries.

## Data Availability

The data presented in this study are available on request from the corresponding author.

## References

[1] Valverde M. (2012). Everyday Law on the Street: City Governance in An Age of Diversity.

[2] Rock M.J. (2013). Pet bylaws and posthumanist health promotion: A case study of urban policy. Crit. Public Health.

[3] Rock M.J., Degeling C., Graham T.M., Toohey A.M., Rault D., McCormack G.R. (2016). Public engagement and community participation in governing urban parks: A case study in changing and implementing a policy addressing off-leash dogs. Crit. Public Health.

[4] Rock M.J., Rault D., Degeling D. (2017). Dog-bites, rabies and One Health: Towards improved coordination in research, policy and practice. Soc. Sci. Med..

[5] Rock M., Degeling C. (2013). Public Health Ethics and a Status for Pets as Person-Things. Bioethical. Inq..

[6] Aronson S. (2010). Animal Control Management: A New Look at A Public Responsibility.

[7] DeMello M. (2021). The Animal Protection Movement. Animals and Society.

[8] Blattner C., Coulter K., Kymlicka W. (2019). Introduction. Animal Labour and the Quest for Interspecies Justice.

[9] Lather P. (1986). Research as Praxis. Harv. Educ. Rev..

[10] Huss R.J. (2008). The Pervasive Nature of Animal Law: How the Law Impacts the Lives of People and Their Animal Companions. Valpso. Univ. Law Rev..

[11] Coulter K. (2022). The Organization of Animal Protection Investigations and the Animal Harm Spectrum: Canadian Data, International Lessons. Soc. Sci..

[12] Rock M.J. (2017). Who or what is ‘the public’ in critical public health? Reflections on posthumanism and anthropological engagements with One Health. Crit. Public Health.

[13] Reese L.A., Remer K.M. (2017). Best Practices in Local Animal Control Ordinances. State Local Gov. Rev..

[14] Arluke A., Patronek G., Lockwood R., Cardona A. (2017). Animal hoarding. Palgrave International Handbook of Animal Abuse Studies.

[15] Campbell K.M. (2013). The Paradox of Animal Hoarding and the Limits of Canadian Criminal Law. J. Anim. Nat. Resour. Law.

[16] Lockwood R. (2018). Animal hoarding: The challenge for mental health, law enforcement, and animal welfare professionals. Behav. Sci. Law.

[17] Timmermans S., Gabe J. (2002). Introduction: Connecting criminology and sociology of health and illness. Sociol. Health Illn..

[18] Coulter K., Fitzgerald A. Difference Makers: Understanding and Improving the OSPCA’s Animal Cruelty Investigation Work 2016. https://humanejobsdotorg.files.wordpress.com/2016/04/difference-makers-understanding-and-improving-the-ospcas-animal-cruelty-investigation-work.pdf.

[19] Arluke A. (2004). Brute Force: Animal Police and the Challenge of Cruelty.

[20] Holmberg T. (2014). Sensuous governance: Assessing urban animal hoarding. Hous. Theory Soc..

[21] Suchman A.L., Sluyter D.J., Williamson P.R. (2011). Leading Change in Healthcare—Transforming Organizations Using Complexity, Positive Psychology and Relationship-centered Care. Leadersh. Health Serv..

[22] Relational Coordination Collaborative. https://relationalcoordination.org.

[23] Gittell J.H., Kyriakidou O., Ozbilgin M. (2006). Relational coordination: Coordinating work through relationships of sharedgoals, shared knowledge and mutual respect. Relational Perspectives in Organizational.

[24] Rault D., Nowicki S., Adams C., Rock M. (2018). To Protect Animals, First We Must Protect Law Enforcement Officers. J. Anim. Nat. Resour. Law.

[25] Townsend A. (2013). Action Research: The Challenges of Understanding and Changing Practice.

[26] Yin R.K. (2013). Case Study Research: Design and Methods.

[27] Rault E.D. (2019). Occupational Health and Safety among Officers Who Enforce Animal Laws in the Province of Alberta (Canada): An Examination of the Risks and Rewards. Ph.D. Thesis.

[28] Wenger E. (1998). Communities of Practice: Learning, Meaning and Identity.

[29] Spradley J.P. (1979). The Ethnographic Interview.

[30] Emerson R.M., Fretz R.I., Shaw L.L. (2011). Writing Ethnographic Fieldnotes.

[31] Alberta Municipal Population Lists. https://www.alberta.ca/municipal-population-lists.aspx.

[32] QSR International NVIVO. http://www.qsrinternational.com/nvivo/nvivo-products.

[33] Hamilton L., Taylor N. (2012). Ethnography in evolution: Adapting to the animal “other” in organizations. J. Organ. Ethnogr..

[34] Green J., Thorogood N. (2013). Qualitative Methods for Health Research.

[35] Braun V., Clarke V. (2020). One size fits all? What counts as quality practice in (reflexive) thematic analysis?. Qual. Res. Psychol..

[36] Tallberg L., Jordan P.J. (2021). Killing Them ‘Softly’ (!): Exploring Work Experiences in Care-Based Animal Dirty Work. Work Employ. Soc..

[37] Coulter K. (2017). Humane Jobs: A Political Economic Vision for Interspecies Solidarity and Human–Animal Wellbeing. Politics Anim..

[38] Sanders C.R. (2010). Working Out Back: The Veterinary Technician and “Dirty Work”. J. Contemp. Ethnogr..

[39] Coulter K. (2016). Animals, Work, and the Promise of Interspecies Solidarity.

[40] Coulter K., Nicholls B., Fitzgerald A. (2022). Animal protection: Organizational constraints and collaborative opportunities. J. Community Saf. Well-Being.

[41] Coulter K., Rault D. (2019). Opinion: Who will protect animals from abuse in Edmonton?. https://edmontonjournal.com/opinion/columnists/opinion-who-will-protect-animals-from-abuse-in-edmonton.

[42] Edmonton Animal Protection. https://www.edmonton.ca/residential_neighbourhoods/pets_wildlife/animal-protection.aspx.

[43] Criminal Code (RSC: 1985. c C-46).

[44] Fraser B. (2018). Report to the Minister of Justice and Solicitor General Public Fatality Inquiry: Rodney Lazenby. https://open.alberta.ca/publications/fatality-inquiry-lazenby-2018-01-09.

[45] Alberta Government Investigation Report: Peace Officer Altercation with Tenant. https://open.alberta.ca/publications/report-no-f-ohs-028275-ad348.

[46] Government of Canada: Canadian Police Information Centre. http://www.cpic-cipc.ca/index-eng.htm.

[47] Royal Canadian Mounted Police-Police Reporting and Occurrence System (PROS). http://www.rcmp-grc.gc.ca/en/police-reporting-and-occurrence-system-pros.

[48] Frost R., Steketee G., Tolin D. (2012). Diagnosis and assessment of hoarding disorder. Annu. Rev. Clin. Psychol..

[49] Valverde M. (2011). Seeing like a city: The dialectic of modern and premodern ways of seeing in urban governance. Law Soc. Rev..

[50] Animal Protection Act.

[51] Hollway J., Grunwald B. (2019). Applying sentinel event reviews to policing. Criminol. Public Policy.

[52] Gnoni M.G., Saleh J.H. (2017). Near-miss management systems and observability-in-depth: Handling safety incidents and accident precursors in light of safety principles. Saf. Sci..

[53] Campbell A.M., Thompson S.L., Harris T.L., Wiehe S.E. (2018). Intimate partner violence and pet abuse: Responding law enforcement officers’ observations and victim reports from the scene. J. Interpers. Violence.

[54] Zilney L.A., Zilney M. (2005). Reunification of child and animal welfare agencies: Cross-reporting of abuse in Wellington County, Ontario. Child Welf..

[55] Mills D.S., Westgarth C. (2017). Dog Bites: A Multidisciplinary Perspective.

[56] Ascione F.R., McDonald S.E., Tedeschi P., Williams J.H. (2018). The relations among animal abuse, psychological disorders, and crime: Implications for forensic assessment. Behav. Sci. Law.

[57] Lipsky M. (2010). Street-Level Bureaucracy: Dilemmas of the Individual in Public Service.

[58] Coulter K., Fitzgerald A. (2017). The compounding feminization of animal cruelty investigation work and its multispecies implications. Gend. Work Organ..

[59] Humane Canada https://humanecanada.ca/team-members/.

[60] Straus S., Tetroe J., Graham I. (2013). Knowledge Translation in Health Care: Moving from Evidence to Practice.

[61] Labrecque J., Walsh C.A. (2011). Homeless women’s voices on incorporating companion animals into shelter services. Anthrozoos Multidiscip. J. Interact. People Anim..

[62] Lockwood R., Arkow P. (2016). Animal abuse and interpersonal violence: The cruelty connection and its implications for veterinary pathology. Vet. Pathol..

[63] Patronek G., Ayers C.R., Steketee G., Frost R.O. (2014). Animal hoarding. The Oxford Handbook of Hoarding and Acquiring.

[64] Stevenson R., Fitzgerald A.J., Barrett B.J. (2018). Keeping pets safe in the context of intimate partner violence: Insights from domestic violence shelter staff in Canada. Affilia.

[65] Burke D. (2017). SPCA Officers Don Body Armour as Animal Cruelty Prosecutions Mount, CBC. http://www.cbc.ca/news/canada/nova-scotia/spca-officers-animal-cruelty-charges-crime-armour-1.3985651.

[66] Coulter K., Campbell B. (2020). Public investment in animal protection work: Data from Manitoba, Canada. Animals.

